# Supplementary material for: NSD1 supports cell growth and regulates autophagy in HPV-negative head and neck squamous cell carcinoma
Source: bioRxiv. 2023 Sep 22:2023.09.19.558537. Preprint. [Version 1] doi: 10.1101/2023.09.19.558537 (PMC10541623; doi:10.1101/2023.09.19.558537)
Supplement: Supplement 1 [file media-1.pdf]

Supplementary Table 1. Cell lines characterization

cell line	histology type	mutations	HPV status
FaDu	Hypopharynx squamous cell carcinoma	*TP53/CDKN2A*	negative (-)
Cal27	Tongue squamous cell carcinoma	*TP53/CDKN2A*	negative (-)
SCC61	Tongue squamous cell carcinoma	*TP53*	negative (-)
SCC4	Tongue squamous cell carcinoma	*TP53/CDKN2A/NSD1 (S752Lfs*16)*	negative (-)
JHU 022	Laryngeal squamous cell carcinoma	*TP53*	negative (-)
JHU 011	Laryngeal squamous cell carcinoma	*TP53*	negative (-)

Supplementary Table 2. shRNA oligos

NSD1 shRNA oligo 1 fw	CCGG CCGAGACGTCTCAGGTTAATCCTCGAGGATTAACCTGAGACGTCTCGGTTTTT
NSD1 shRNA oligo 1 rev	AATT AAAAACCGAGACGTCTCAGGTTAATCCTCGAGGATTAACCTGAGACGTCTCGG
NSD1 shRNA oligo 2 fw	CCGG AGGAGTGGATGGGACATATAACTCGAGTTATATGTCCCATCCACTCCTTTTTT
NSD1 shRNA oligo 2 rev	AATT AAAAAAGGAGTGGATGGGACATATAACTCGAGTTATATGTCCCATCCACTCCT

Supplementary Table 3. siRNA sequences

Gene symbol	Sequence	Supplier	Catalog number
NSD1 (si1)	5`-CUCAAGAACAUGAUAUCACUAAUTT-3`	Integrated DNA Technologies	#402447171
	3`-ACGAGUUCUUGUACUAUAGUGAUUAAA-5`		(hs.Ri.NSD1.13.1)
NSD1 (si2)	5`-GUUAAAAUCAUGAAAGCAGUCACTA -3`	Integrated DNA Technologies	#402447174
	3`-ACCAAUUUUAGUACUUUCGUCAGUGAU-5`		(hs.Ri.NSD1.13.2)

Supplementary Table 4. PCR primer sequences

Gene symbol	Primer sequence		Supplier	RefSeqNumber
NSD1	Primer 1	5'-AGCTCGTCTCCTGCAAGA-3'	Integrated DNA Technologies	NM_ 172349
	Primer 2	5'-CAGATGTCACACTGATGCCA-3'		
WHSCl (NSD2)	Primer 1	5'-TCGGAAGAGAGACACAATCAC-3'	Integrated DNA Technologies	NM_ 133335
	Primer 2	5'-GTGGTTTACATGCATCAGACAG-3'		
WHSC1L1 (NSD3)	Primer 1	5'-GTATCATCTCCTGAAGCAACATC-3	Integrated DNA Technologies	NM_023034
	Primer 2	5'-GAACTGTTTCAACCTGCTCCT-3'		
ULK1	Primer 1	5'-CTACCTGGTTATGGAGTACTGC-3'	Integrated DNA Technologies	NM_003565
	Primer 2	5'-GGAAGAGCCTGATGGTGTC-3'		
AKT	Primer 1	5'- CTCCCCTCAACAACTTCTCTG-3'	Integrated DNA Technologies	NM_005163
	Primer 2	5'- GCGTTCGATGACAGTGGT-3'		
RNA18S5	Primer 1	5'-GAGACTCTGGCATGCTAACTAG-3'	Integrated DNA Technologies	NR_003286
	Primer 2	5'-GGACATCTAAGGGCATCACAG-3'		

Supplementary Table 5. Antibodies and dilutions

Antibody name	Assay (IHC, Western blot, IHC)	Supplier, catalog number	Dilution
**Primary antibodies**			
NSD1	Western blot	NeuroMab, 75-280	1:750
NSD1	IHC	Invitrogen, #PA5-84938	1:500
WHSC1/NSD2	Western blot	Abcam, ab75359	1:1000
WHSC1L1 (NSD3)	Western blot	Cell Signaling Technology, #92056	1:1000
Di-Methyl-Histone H3 (Lys36)	Western blot/IHC	Cell Signaling Technology, #2901	1:1000/1:200
Histone H3	Western blot	Cell Signaling Technology, #14269	1:1000
Phospho-mTOR (Ser2448)	Western blot	Cell Signaling Technology, #2971	1:1000
mTOR	Western blot	Cell Signaling Technology, #4517	1:500
Phospho-p70 S6 Kinase (Thr389)	Western blot	Cell Signaling Technology, #9205	1:1000
p70 S6 Kinase	Western blot	Cell Signaling Technology, #34475	1:1000
Phospho-S6 Ribosomal Protein (Ser240/244)	Western blot	Cell Signaling Technology, #2215	1:1000
S6 Ribosomal Protein	Western blot	Cell Signaling Technology, #2217	1:1000
Phospho-Akt (Ser473)	Western blot	Cell Signaling Technology, #4069	1:2000
Phospho-Akt (Thr308)	Western blot	Cell Signaling Technology, #4056	1:1000
Akt (pan)	Western blot	Cell Signaling Technology, #2920	1:2000
Phospho-Tuberin/TSC2 (Thr1462)	Western blot	Cell Signaling Technology, #3617	1:1000
Tuberin/TSC2	Western blot	Cell Signaling Technology, #4308	1:1000
Phospho-p44/42 MAPK (Erk1/2) (Thr202/Tyr204	Western blot	Cell Signaling Technology, #4370	1:2000
p44/42 MAPK (Erk1/2)	Western blot	Cell Signaling Technology, #9201	1:1000
ULK1	Western blot	Cell Signaling Technology, #6439	1:700
SQSTM1/p62	Western blot	Cell Signaling Technology, #5114	1:1000
LC3A/B	Western blot	Cell Signaling Technology, #12741	1:1000
Phospho-AMPKα (Thr172)	Western blot	Cell Signaling Technology, #50081	1:1000
AMPKα	Western blot	Cell Signaling Technology, #2793	1:700
Phospho-Beclin-1 (Ser30)	Western blot	Cell Signaling Technology, #35955	1:1000
Phospho-Beclin-1 (Ser93)	Western blot	Cell Signaling Technology, #14717	1:1000
Beclin-1	Western blot	Cell Signaling Technology, #4122	1:700
Vinculin	Western blot	Cell Signaling Technology, #13901	1:500
p62	IHC	Novus Bio, NBP1-48320	1:600
LC3B	IHC	Novus Bio, NB100-2220	1:600
**Secondary antibodies**			
Anti-rabbit IgG, HRP-linked Antibody	Western blot	Cell Signaling Technology, #7074	1:1500
Anti-mouse IgG, HRP-linked Antibody	Western blot	Cell Signaling Technology, #7076	1:1500

Supplementary Table 6. Patient characteristics for the primary HNSCC tumors

**Anatomical site**	**%**
Larynx	11
Lymph node	3
Neck	11
Tongue	67
Tonsil	8
**Prognostic stage**	**%**
I	0
II	11
III	36
IVa	19
IVb	33
**T stage**	**%**
Tx	2
T1	8
T2	19
T3	44
T4	25
**Lymph nodes**	**%**
Negative	36
Positive	64

TMAs contained specimens from 36 patients (with characteristics --noted in the table), and for 6 normal epithelial tissues.

Table 7. PCR primer sequences for ChIP-assay

Gene symbol	Primer sequence		Supplier
ULK1(-1225…-1142)	Primer 1	5'- CGTGTACGGTGAACAGCACT-3'	Integrated DNA Technologies
	Primer 2	5'- GGGCTCACTCACAGAAGACA-3'	
ULK1 (-636…-552)	Primer 1	5'-CCTAACCTCTAACTCAGCCATTCT-3'	Integrated DNA Technologies
	Primer 2	5'-GAGAACAGGCGTGGGAAATG-3'	
ULK1 (+1038…+1175)	Primer 1	5'-CAAAATCCTGAAGGTGAGCCAG-3	Integrated DNA Technologies
	Primer 2	5'-GTCGTACAGGGCCACGATG-3'	
ULK1 (+15 319…+15 418)	Primer 1	5'-GCCCTACTGCAACGCAAC-3'	Integrated DNA Technologies
	Primer 2	5'-ATCAGCAAATGCAAGGAAGGAG-3'	
ULK1 (+28 439…+ 28 540)	Primer 1	5'-GTGTGATTTCCTGCCCTTTGC-3'	Integrated DNA Technologies
	Primer 2	5'-CAAGCCAACGAAGACAAGTGG-3'	
