# Supplementary figures and images for: NSD1 supports cell growth and regulates autophagy in HPV-negative head and neck squamous cell carcinoma
Source: bioRxiv. 2023 Sep 22:2023.09.19.558537. Preprint. [Version 1] doi: 10.1101/2023.09.19.558537 (PMC10541623; doi:10.1101/2023.09.19.558537)

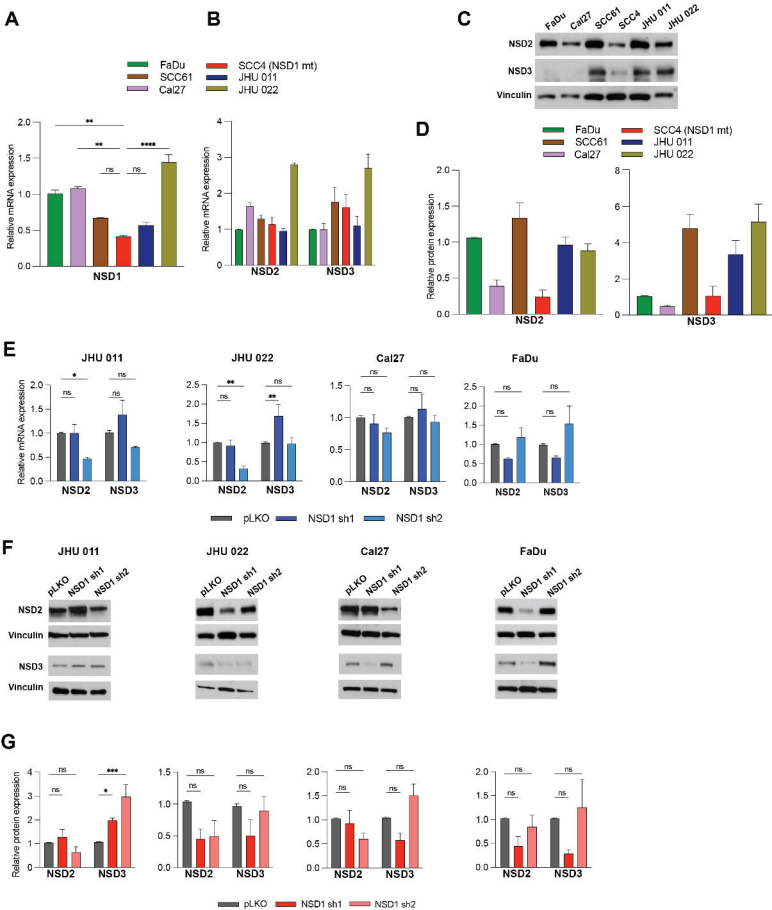

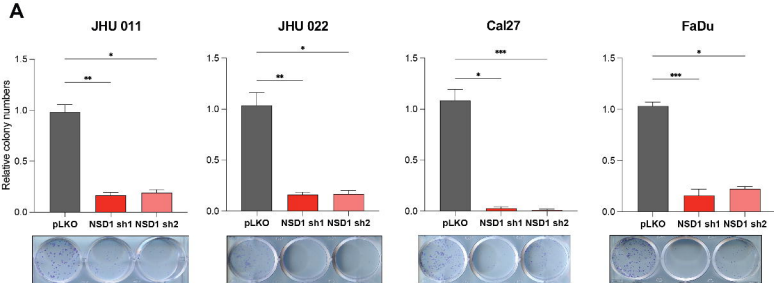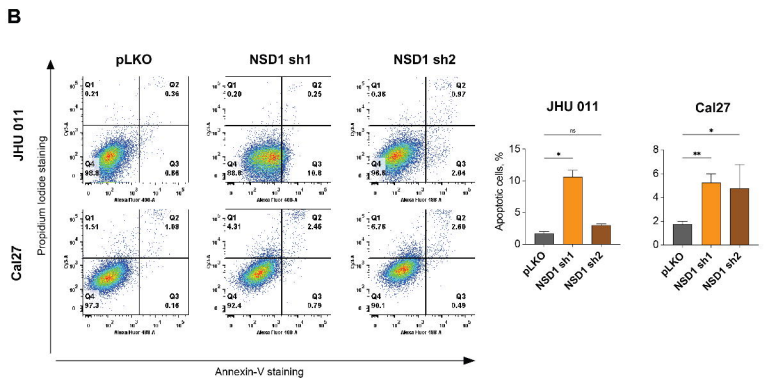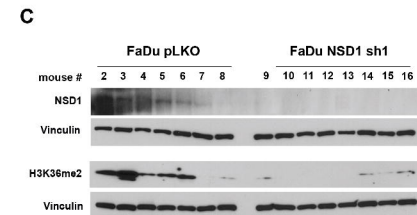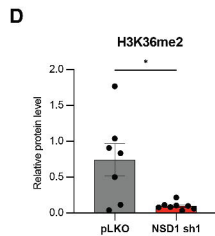

# Cellular Response to Starvation

# Autophagosome

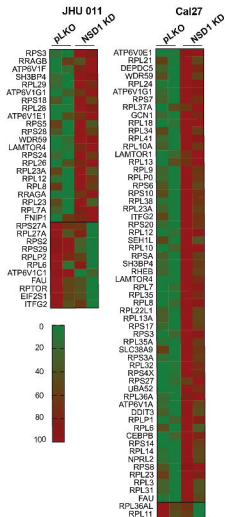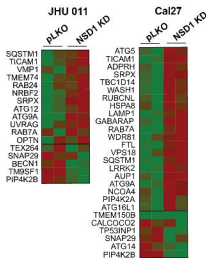

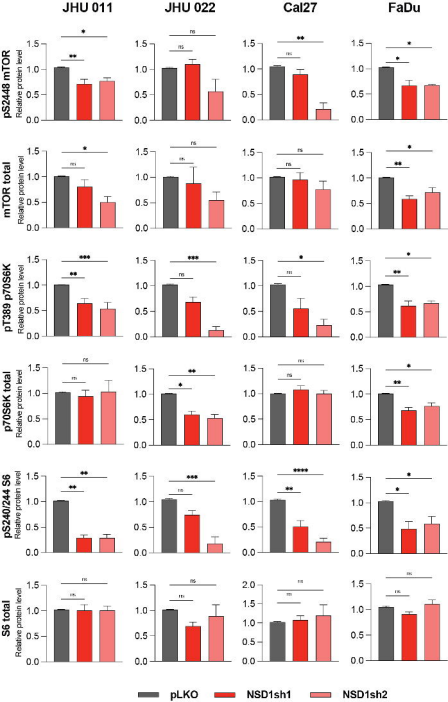

Supplement: Supplement 2 — Supplementary Figure S1 (A) Relative mRNA level of NSD1 gene, measured by RT-qPCR in human HNSCC cell lines. NSD1 relative level was normalized on 18S as a control gene. Statistical significance was determined by Kruskal-Wallis with Dunn’s multiple comparisons post-test. (B) Relative mRNA level of NSD2 and NSD3 genes was measured by RT-qPCR in head and cancer cell lines. NSD2 and NSD3 relative level were normalized to 18S as a control gene. (C) Western blot of NSD2 and NSD3 protein levels in a panel of human HNSCC cell lines. (D) Quantification of Western blot images in (C). (E) mRNA level of NSD2 and NSD3 genes was measured by RT-qPCR after NSD1 shRNA knockdown cells at 72h after knockdown induction. NSD2 and NSD3 relative level were normalized to 18S as a control gene. Statistical significance determined by ANOVA with Dunnett multiple comparison post-test. (F) Western blot of NSD2 and NSD3 protein levels upon induction of pLKO control or NSD1 shRNA knockdown at 72h with doxycycline (G) Quantification of Western blot images in (F). Statistical significance was determined by ANOVA with Dunnett multiple comparison post-test. Experiments were performed in at least three independent biological repeats. The error bars are presented as mean ± SEM. ns – not significant, *p<0.05, **p<0.01, ***p<0.001, and ****p<0.0001. Supplementary Figure S2 (A) Representative images of colony formation assay and quantification of the relative colony numbers in a panel of human HNSCC cell lines. Statistical significance was determined by Kruskal-Wallis with Dunn’s multiple comparisons post-test. (B) Representative flow cytometry images (left) and average calculations (right) of the Annexin V/Propidium iodide (PI) staining as measured by flow cytometry at 144h after NSD1 knockdown induction in JHU 011 and Cal27 cell lines. Statistical significance was determined by Kruskal-Wallis with Dunn’s multiple comparisons post-test. (C) Western blot of NSD1 and H3K36me2 levels in mice tumors. Mouse [file NIHPP2023.09.19.558537v1-supplement-2.pdf]
